# Rhegmatogenous Retinal Detachment Associated With a Retinal Tear Overlying a Choroidal Nevus

**DOI:** 10.1155/crop/6311293

**Published:** 2026-05-05

**Authors:** Georgios Mylonas, Gregor S. Reiter, Markus Ritter, Gabor G. Deak, Adrian Reumueller, Andreas Pollreisz, Stefan Sacu, Michael Georgopoulos

**Affiliations:** ^1^ Department of Ophthalmology, Medical University of Vienna, Vienna, Austria, meduniwien.ac.at

## Abstract

**Purpose:**

The purpose of the study is to report a case of a rhegmatogenous retinal detachment (RRD) associated with a retinal tear overlying a choroidal nevus.

**Methods:**

This study is a case report.

**Results:**

A 66‐year‐old female patient has been referred to our department because of a retinal detachment in her left eye. Her ocular history was notable for a choroidal nevus in her left eye, which has been under observation for the past 11 years without any signs of malignancy or abnormal changes. Dilated funduscopic examination revealed a superior macula‐off bullous RRD that originated from a retinal tear located at the previously observed choroidal nevus. The patient successfully underwent a 23‐g vitrectomy and cryotherapy with SF6 gas tamponade, combined with cataract surgery and biopsy.

**Conclusion:**

While choroidal nevi are relatively prevalent, the specific progression to RRD due to a tear is a rare occurrence in clinical practice, and it presents with unique diagnostic and therapeutic challenges.

## 1. Introduction

Rhegmatogenous retinal detachment (RRD) is the most common form of retinal detachment, affecting approximately 1 in 10,000 people annually [1]. It happens when a “break” or full‐thickness defect develops in the neurosensory retina, allowing fluid from the vitreous cavity to seep into the subretinal space. These results in the separation of the neurosensory retina from the underlying retinal pigment epithelium [2]. Retinal breaks commonly occur at the posterior border of the vitreous base and can happen in any quadrant, with the most frequent location being the superior temporal quadrant (60%), followed by the superior nasal quadrant.

Choroidal nevus is the most common benign intraocular tumor, found predominantly in Whites [3]. They are small melanocytic lesions and have been shown to be rather evenly distributed in the posterior fundus, with a predilection for both the nasal and temporal quadrants [4]. The incidence of choroidal nevi varies by population and study, and it is approximately 4%–6.5% [5–7].

While choroidal nevi are relatively common, the coexistence of RRD and a retinal tear overlying a choroidal nevus is an uncommon finding in clinical practice and may pose unique diagnostic and therapeutic challenges. The presence of a choroidal nevus in proximity to a retinal break can complicate the diagnosis of RRD, as distinguishing features of benign lesions versus malignant transformation or retinal tears may overlap. Accurate diagnosis is crucial in these cases, as the management of RRD in patients with concurrent choroidal nevi requires careful consideration to avoid complications and optimize visual outcomes.

## 2. Case Presentation

A 66‐year‐old female patient presented to our department with a retinal detachment in her left eye. She reported experiencing a reduction in her visual field for the past 3 days and significant visual impairment over the last 2 days, specific to her left eye. Her ocular history was remarkable for a choroidal nevus in her left eye, which had been regularly monitored for the past 11 years. Throughout this period, the nevus showed no signs of malignancy or abnormal progression. No written consent has been obtained from the patients, as there is no patient‐identifiable data included in this case report.

**Figure 1:**
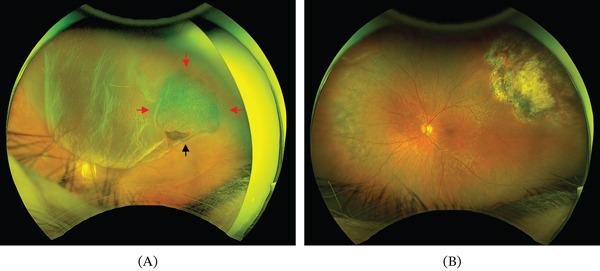
(A) Preoperative ultrawidefield image showing the bullous rhegmatogenous retinal detachment. The retinal tear is indicated by the black arrow and is located within a choroidal nevus, marked by red arrows. (B) Postoperative ultrawidefield image showing the reattached retina and the cryotherapy scar over the area of the previous choroidal nevus.

**Figure 2:**
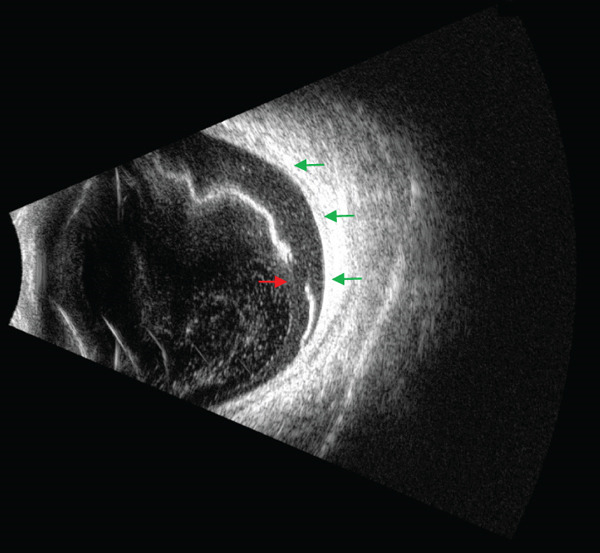
Preoperative ultrasound image of the rhegmatogenous retinal detachment, showing the retinal tear (red arrow) and a flattened choroid (green arrows) beneath the detachment, with no indications of malignancy or active tumor growth.

Upon examination, her visual acuity in the affected eye was found to be not quantifiable, with only hand motion detection possible at a 2‐m distance. A slit‐lamp examination indicated normal anterior segments except for a mild nuclear cataract. However, a dilated funduscopic exam revealed a superior macula‐off bullous RRD extending from a retinal tear, precisely located over the area of the previously observed choroidal nevus (Figure 1A). During funduscopy, visualization of the choroidal lesion was limited due to the detached retina. To further assess the choroidal structure and rule out potential complications, such as choroidal melanoma, an ocular ultrasound was performed. The examination revealed a flattened choroid with no signs of malignancy, such as tumor prominence extending into the vitreous cavity (Figure 2).

Given the location of the tear and its association with the choroidal nevus, a comprehensive treatment plan was devised to address both the RRD and the nevus. The patient underwent a 23‐G pars plana vitrectomy combined with cryotherapy and SF6 gas tamponade. In addition, cataract surgery and implantation of a clear monofocal hydrophobic acrylate lens into the capsular bag were performed to address the mild cataract identified during the examination. During the vitrectomy, we observed that the area of detached retina over the choroidal nevus was slightly pigmented. To rule out any potential malignancy, a retinal biopsy of the detached tissue in this area was also performed. Cryotherapy was carefully targeted to the area around the nevus as an added precaution, should histopathological analysis reveal any malignancy.

In the 3‐month follow‐up examination, the patient′s visual acuity had improved to 0.8 (Snellen^′^s equivalent≙0.1 logMAR). The anterior segment examination was unremarkable, and the retina remained firmly attached with no signs of recurrent detachment. A cryotherapy scar was visible over the area of the previous choroidal nevus, marking the treated site and providing a clear indicator of the therapeutic intervention (Figure 1B). Histopathological examination of the biopsy specimen identified no malignant cells, further supporting the benign nature of the choroidal nevus and minimizing concern for malignancy.

## 3. Discussion

To our knowledge, this is the first case report describing an RRD associated with a retinal tear overlying a choroidal nevus, representing an uncommon clinical scenario that highlights the coexistence of benign ocular lesions and retinal pathology. Although choroidal nevi are typically stable, asymptomatic, and benign lesions, vitreoretinal tractional complications such as retinal tears or RRD are not established features of their natural history. Nevertheless, this case highlights that they may still give rise to diagnostic and therapeutic dilemmas in certain clinical scenarios.

Compared to RRD with coexisting choroidal melanoma, the link between RRD and choroidal nevi is poorly documented. Studies of melanoma cases indicate that multiple surgeries are often necessary for retinal reattachment; however, visual outcomes remain poor due to the tumor itself and its treatment. Vitrectomy appears to be safe with regard to tumor spread in previously treated cases [8]. When RRD is accompanied by suspicious choroidal elevation, the possibility of choroidal melanoma should be considered in the differential diagnosis [9].

In the case discussed, the patient′s choroidal nevus had been observed for over a decade without showing malignant characteristics, highlighting the typically benign course of such nevi. This stability over time, however, does not negate the importance of ongoing vigilance. Imaging techniques, particularly ocular ultrasound, were pivotal in this case, enabling a clear assessment of the choroid and adjacent structures. The ultrasound findings of a flattened choroid with no evidence of melanoma provided reassurance that the nevus itself had not transformed malignantly. This scenario demonstrates that a thorough assessment of choroidal and retinal status can aid in distinguishing between a stable nevus and more concerning, potentially malignant features.

The management strategy in this case included several crucial components: vitrectomy, cryotherapy, and a biopsy of the detached retinal tissue adjacent to the nevus. The biopsy offered a histopathological examination of the affected tissue, which is especially valuable in cases where malignancy might be suspected. While choroidal nevi rarely undergo malignant transformation, a biopsy can provide definitive evidence to rule out malignancy when clinical suspicion arises due to changes in the lesion or associated retinal abnormalities. Although the negative biopsy result supported the presumed benign nature of the lesion and allowed management to focus on repair of the RRD without additional oncologic intervention, malignancy cannot be definitively excluded. The biopsy specimen was obtained from retinal tissue rather than the choroid, the structure most appropriate for assessing a suspected choroidal lesion; therefore, the result should be interpreted with caution. Malignant cells would likely have been identified only in the presence of tumor extension from the choroid into the overlying retina. A direct choroidal biopsy was not pursued due to the flat morphology of the nevus and the potential risk of procedural complications.

The choice to perform vitrectomy with SF6 gas tamponade combined with cryotherapy reflects a comprehensive approach aimed at both reattaching the retina and securing the area around the choroidal nevus to prevent further complications. The cryotherapy was applied to the nevus area as a precaution, considering the possibility—though low—of malignant potential, which highlights the importance of conservative measures in cases of uncertain risk.

This case further underscores the importance of early and aggressive intervention when retinal detachment is detected in proximity to a choroidal nevus. Postoperative outcomes in this case were positive, with a notable recovery in visual acuity and an attached retina during follow‐up. This outcome illustrates that a tailored, proactive approach to both retinal detachment and the presence of a choroidal nevus can result in favorable patient outcomes.

Future research is needed to better understand the risk factors associated with retinal tears and RRD in patients with choroidal nevi. Although choroidal nevi are common, large‐scale epidemiological studies could help identify specific factors that increase the likelihood of retinal detachment in these patients, such as the size, location, or growth patterns of the nevi. Additionally, advancements in imaging modalities, including enhanced‐depth imaging optical coherence tomography (EDI‐OCT) and other noninvasive techniques, may improve clinicians′ ability to assess subtle changes in choroidal nevi over time, potentially allowing for earlier intervention if any risk indicators are identified.

This case highlights the significance of a comprehensive approach to managing RRD associated with a choroidal nevus. Vigilant monitoring of choroidal nevi, careful consideration of potential complications, and prompt surgical intervention when RRD develops are essential to preserving vision in these patients. Clinicians should be aware of the possible complications even in long‐standing benign nevi and should consider the value of targeted imaging and histopathological analysis when RRD occurs in these cases. Future studies may elucidate more precise guidelines for managing these patients, with the aim of preventing retinal detachment and ensuring the long‐term stability of both the retina and the choroidal nevus.

## Funding

Open access funding is provided by Medizinische Universitat Wien.

## Conflicts of Interest

The authors declare no conflicts of interest.

## Data Availability

The data that support the findings of this study are available on request from the corresponding author. The data are not publicly available due to privacy or ethical restrictions.
